# Improved Preservation of Residual Beta Cell Function by Atorvastatin in Patients with Recent Onset Type 1 Diabetes and High CRP Levels (DIATOR Trial)

**DOI:** 10.1371/journal.pone.0033108

**Published:** 2012-03-20

**Authors:** Alexander Strom, Hubert Kolb, Stephan Martin, Christian Herder, Marie-Christine Simon, Wolfgang Koenig, Tim Heise, Lutz Heinemann, Michael Roden, Nanette C. Schloot, on behalf of the DIATOR Study Group

**Affiliations:** 1 Institute for Clinical Diabetology, German Diabetes Center, Leibniz Center for Diabetes Research at Heinrich Heine University, Düsseldorf, Germany; 2 Immunobiology Research Group, University Hospital, University of Düsseldorf, Düsseldorf, Germany; 3 West-German Centre of Diabetes and Health, Verbund Katholischer Kliniken Düsseldorf, Düsseldorf, Germany; 4 Department of Internal Medicine II - Cardiology, University of Ulm Medical Center, Ulm, Germany; 5 Profil Institute for Metabolic Research, Neuss, Germany; 6 Department of Metabolic Diseases, University Hospital, Düsseldorf, Germany; La Jolla Institute for Allergy and Immunology, United States of America

## Abstract

**Background:**

A recent randomized placebo-controlled trial of the effect of atorvastatin treatment on the progression of newly diagnosed type 1 diabetes suggested a slower decline of residual beta cell function with statin treatment. Aim of this secondary analysis was to identify patient subgroups which differ in the decline of beta cell function during treatment with atorvastatin.

**Methodology/Principal Findings:**

The randomized placebo-controlled Diabetes and Atorvastatin (DIATOR) Trial included 89 patients with newly diagnosed type 1 diabetes and detectable islet autoantibodies (mean age 30 years, 40% females), in 12 centers in Germany. Patients received placebo or 80 mg/d atorvastatin for 18 months. As primary outcome stimulated serum C-peptide levels were determined 90 min after a standardized liquid mixed meal. For this secondary analysis patients were stratified by single baseline characteristics which were considered to possibly be modified by atorvastatin treatment. Subgroups defined by age, sex or by baseline metabolic parameters like body mass index (BMI), total serum cholesterol or fasting C-peptide did not differ in C-peptide outcome after atorvastatin treatment. However, the subgroup defined by high (above median) baseline C-reactive protein (CRP) concentrations exhibited higher stimulated C-peptide secretion after statin treatment (p = 0.044). Individual baseline CRP levels correlated with C-peptide outcome in the statin group (r^2^ = 0.3079, p<0.004). The subgroup with baseline CRP concentrations above median differed from the corresponding subgroup with lower CRP levels by higher median values of BMI, IL-6, IL-1RA, sICAM-1 and E-selectin.

**Conclusions/Significance:**

Atorvastatin treatment may be effective in slowing the decline of beta cell function in a patient subgroup defined by above median levels of CRP and other inflammation associated immune mediators.

**Trial Registration:**

ClinicalTrials.gov NCT00974740

## Introduction

Treatment with statins has been found to dampen inflammatory reactions and immune activation in general, and some positive results have also been reported for intervention trials in rheumatoid arthritis [1], [2], [3]. Most studies of statin treatment in animal models of immune destruction of beta cells also observed some protection of beta cells or improved regeneration [4], [5], [6], [7], [8].

In a recent trial of atorvastatin in patients with newly diagnosed type 1 diabetes (Diabetes and Atorvastatin, DIATOR) the primary analysis did not show a significant effect of statin treatment on the progressive loss of beta cell function at 18 months, as determined from serum C-peptide concentrations after a standardized liquid mixed meal [9]. However, descriptive analyses suggested a slower decline of fasting and meal-stimulated C-peptide concentrations in patients of the atorvastatin group, suggesting better preservation of beta-cell function over the 18 months of the trial. We therefore performed a secondary analysis of the data set in order to identify a patient subgroup with improved preservation of residual C-peptide secretion in response to atorvastatin treatment.

## Results

Patients were stratified by single baseline characteristics which were considered to possibly associate with atorvastatin treatment. These characteristics comprised basic anthropomorphic, metabolic and immune parameters. For each parameter patients with baseline values at or below the median were compared with those above the median. Alternatively, patients were stratified according to sex. Pre-defined primary outcome measure was the median stimulated C-peptide concentration at 18 months. In the placebo group, C-peptide outcome was dependent on some baseline metabolic parameters, i.e., significantly higher median stimulated C-peptide concentrations at 18 months were observed in subgroups defined by lower BMI, higher fasting or higher stimulated C-peptide levels at baseline (**Table 1**). In the atorvastatin group, there was less association with baseline metabolic parameters, only higher stimulated C-peptide secretion at baseline predicted better C-peptide outcome at 18 months. Of two clinically relevant targets of statin action, total cholesterol and CRP, baseline CRP levels associated with C-peptide outcome. This means there was a lower decline of C-peptide secretion in the subgroup with higher baseline CRP concentrations. No association with outcome was seen for patient subgroups defined by higher vs. lower baseline IL-6 concentrations (**Table 1**). For each single characteristic association with outcome was calculated with adjustments for all other baseline parameters indicated in the table.

**Table 1:** Baseline characteristics of patients versus outcome.

Placebo				Atorvastatin			
Baseline characteristic		C-peptide*	*P*-value	Baseline characteristic		C-peptide*	*P*-value
Age	below median	0.36 [0.58]	n.s.	Age	below median	0.53 [0.87]	n.s.
	above median	0.69 [0.92]			above median	0.72 [0.70]	
Sex	male	0.42 [0.80]	n.s.	Sex	male	0.73 [0.94]	n.s.
	female	0.57 [0.77]			female	0.58 [0.98]	
BMI	below median	0.81 [0.68]	0.023	BMI	below median	0.73 [0.96]	n.s.
	above median	0.29 [0.34]			above median	0.67 [0.92]	
Fasting C-peptide	below median	0.40 [0.46]	0.011	Fasting C-peptide	below median	0.27 [0.74]	n.s.
	above median	0.85 [1.17]			above median	0.82 [0.69]	
Stimulated C-peptide	below median	0.34 [0.36]	0.0007	Stimulated C-peptide	below median	0.26 [0.72]	0.031
	above median	0.93 [1.05]			above median	0.82 [0.66]	
Total cholesterol	below median	0.50 [0.79]	n.s.	Total cholesterol	below median	0.79 [0.87]	n.s.
	above median	0.40 [0.84]			above median	0.69 [0.70]	
CRP	below median	0.66 [0.65]	n.s.	CRP	below median	0.40 [0.75]	0.044
	above median	0.42 [1.18]			above median	0.76 [0.76]	
IL-6	below median	0.40 [0.62]	n.s.	IL-6	below median	0.73 [0.86]	n.s.
	above median	0.50 [1.07]			above median	0.58 [0.76]	

* - the column shows stimulated C-peptide concentrations at 18 months (nmol/l) [IQR].

Data are presented as median concentrations and interquartile range [IQR].

**Figure 1:**
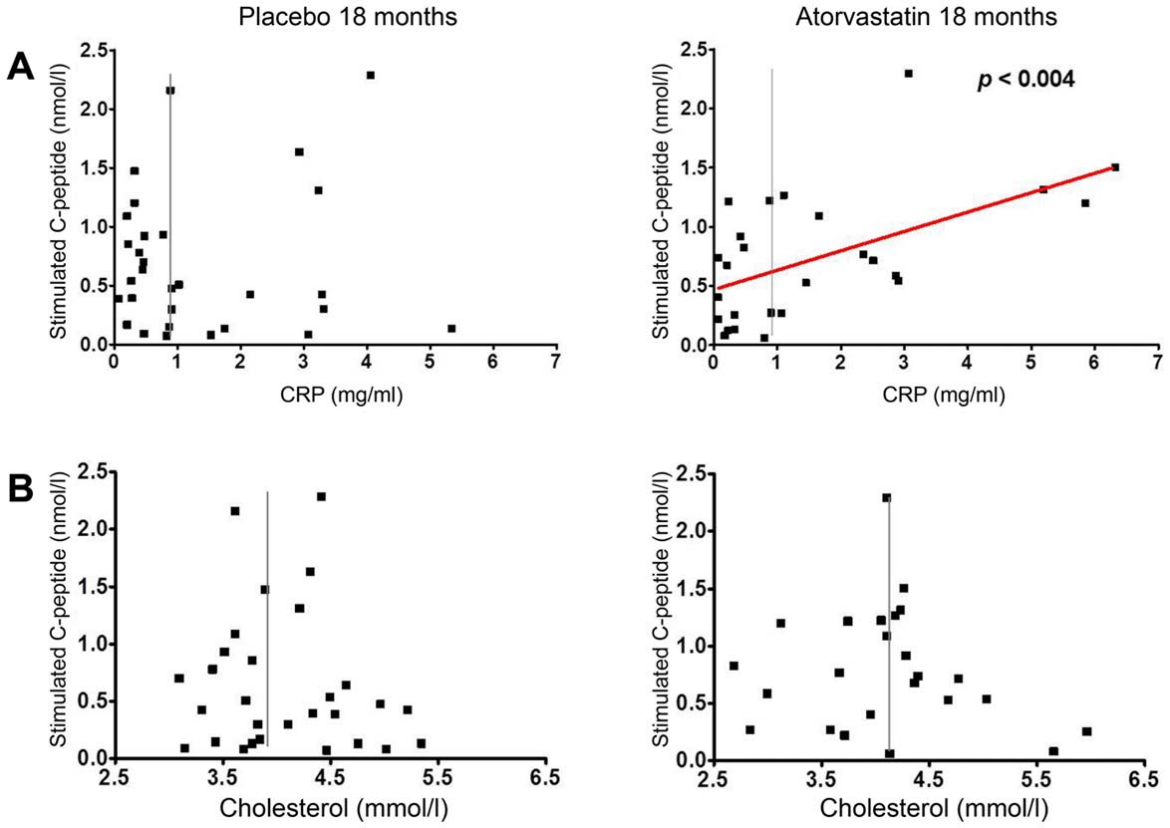
Correlation analysis of baseline CRP or total serum cholesterol concentrations with C-peptide outcome. Of individual patients, baseline CRP concentrations (**a**) or baseline total cholesterol levels (**b**) were compared to stimulated C-peptide concentrations at 18 months (18 m). The vertical line indicates the median of baseline CRP or cholesterol concentrations, respectively, the red line indicates the linear regression line obtained by the Pearson's correlation test.

To study the association between baseline CRP concentrations and C-peptide outcome in more detail, single patient data are depicted in **Fig. 1** in the format of a Pearson's correlation test. There was a significant linear correlation between baseline CRP concentrations and C-peptide outcome in the statin group but not the placebo group (r^2^ = 0.3079, p<0.004) (**Fig. 1A**). Only two out of 13 patients with high CRP baseline levels had stimulated C-peptide concentrations below the median of the subgroup with low baseline levels (median 0.40 nmol/l). Baseline cholesterol levels and C-peptide outcome were not associated (**Fig. 1B**).

**Figure 2:**
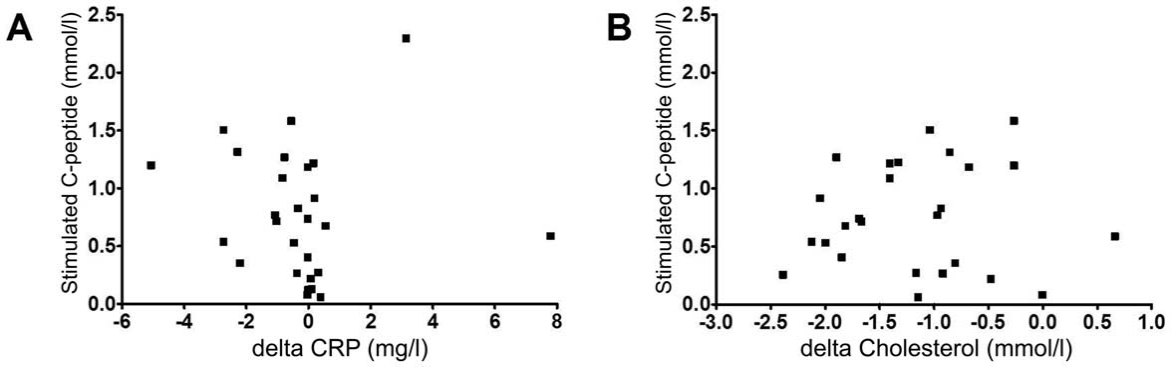
Correlation analysis of atorvastatin effects on cholesterol or CRP levels with C-peptide outcome. Of individual patients, the change of CRP concentrations (**a**) or total cholesterol concentrations (**b**) from baseline to 3 months of atorvastatin treatment (“delta”) were compared to stimulated C-peptide concentrations at 18 months (18 m).

Both targets of statins, total cholesterol and CRP levels, were lowered by atorvastatin treatment, while IL-6 concentrations were not modified (**Table 2**). Total cholesterol levels were decreased in both subgroups, in those with initially high or with initially low total cholesterol levels. CRP levels were reduced (p = 0.037) upon atorvastatin treatment only in the patient subgroup with initially high (above median) CRP concentrations (**Table 2**). However, the extent of CRP level lowering by atorvastatin was not associated with the extent of residual C-peptide secretion after 18 months (**Fig. 2A**). The extent of cholesterol level lowering also did not correlate with C-peptide outcome (**Fig. 2B**).

**Table 2:** Effect of atorvastatin treatment on levels of cholesterol and immune mediators.

Parameter		Placebo (median) [IQR]		Atorvastatin (median) [IQR]	
Total cholesterol (mmol/l)	At baseline	4.02 [1.03]	n.s.	3.93 [0.96]	p<0.0001
	At 3 months	3.89 [1.13]		2.56 [0.78]	
CRP (mg/l)	At baseline	0.84 [2.24]	n.s.	0.86 [2.17]	p = 0.029
	At 3 months	0.64 [1.41]		0.69 [1.07]	
IL-6 (pg/ml)	At baseline	0.58 [0.54]	n.s.	0.75 [1.08]	n.s.
	At 3 months	0.67 [0.50]		0.56 [1.62]	
Cholesterol above baseline median	At baseline	4.53 [0.70]	n.s.	4.33 [0.57]	p<0.0001
	At 3 months	4.44 [0.96]		2.94 [0.61]	
Cholesterol below baseline median	At baseline	3.57 [0.39]	n.s.	3.39 [0.69]	p<0.0001
	At 3 months	3.47 [0.63]		2.35 [0.76]	
CRP above baseline median	At baseline	2.55 [2.75]	n.s.	2.37 [1.90]	p = 0.037
	At 3 months	1.73 [3.39]		1.24 [0.86]	
CRP below baseline median	At baseline	0.33 [0.23]	n.s.	0.24 [0.40]	n.s.
	At 3 months	0.36 [0.31]		0.21 [0.34]	

Since of all parameters analyzed, only baseline CRP levels were associated with preservation of C-peptide secretion in the statin group, we compared the subgroup with higher versus lower baseline CRP concentrations for other characteristics. As shown in **Table 3**, patients with higher CRP levels also exhibited greater median concentrations for the inflammation-associated immune mediators IL-6 (p = 0.021), IL-1RA (p = 0.036), sICAM-1 (p = 0.016) and E-selectin (p = 0.034). However, there was no general immune activation because several other immune mediators did not exhibit increased concentrations in circulation (IFN-γ, IL-18, MCP-1, IP-10, MCP-4, MIP-1ß, MDC, IL-8, TARC). Patients with higher baseline CRP concentrations had higher median BMI and fasting C-peptide levels (**Table 3**).

**Table 3:** Characteristics of atorvastatin subgroups with lower vs. higher baseline CRP levels.

Characteristic	Baseline CRP below median	Baseline CRP above media	P-value
Median age, y [IQR]	27 [12.5]	36 [8.0]	0.0056
Men, n (%)	13 (72)	7 (41)	n.s.
Median BMI (kg/m^2^) [IQR]	22.5 [3.9]	25.6 [4.5]	0.0002
Median fasting C-peptide (nmol/l) [IQR]	0.34 [0.22]	0.46 [0.37]	0.019
Median stim. C-peptide (nmol/l) [IQR]	0.78 [0.63]	1.11 [0.77]	n.s.
IL-6 (pg/ml) [IQR]	0.50 [0.54]	1.01 [0.95]	0.021
IFN-γ (pg/ml) [IQR]	2.53^a^ [2.55]	1.92^b^ [0.89]	n.s.
IL-18 (pg/ml) [IQR]	131 [105]	107 [52]	n.s.
IL-1RA (pg/ml) [IQR]	263 [166]	380 [306]	0.036
MCP-1 (pg/ml) [IQR]	367 [347]	362 [182]	n.s.
IP-10 (pg/ml) [IQR]	150^a^ [82]	144^b^ [57]	n.s.
MCP-4 (pg/ml) [IQR]	319^a^ [180]	363^b^ [140]	n.s.
MIP-1ß (pg/ml) [IQR]	616^a^ [404]	751^b^ [493]	n.s.
MDC (pg/ml) [IQR]	4841^a^ [2137]	5049^b^ [4562]	n.s.
IL-8 (pg/ml) [IQR]	3.00^a^ [2.72]	3.86^b^ [4.72]	n.s.
TARC (pg/ml) [IQR]	465^a^ [411]	305^b^ [307]	n.s.
sICAM-1 (ng/ml) [IQR]	162 [77]	201 [82]	0.016
E-selectin (ng/ml) [IQR]	29.95 [19.95]	39.70 [14.60]	0.034

a Data available for 15 of 18 patients.

b Data available for 15 of 17 patients.

Subgroups were compared for single characteristics without adjustments for differences in other characteristics, in order to describe all differences observed.

## Discussion

The primary analysis of the DIATOR trial did not find a significant effect of atorvastatin treatment on stimulated C-peptide concentrations at 18 months, although median C-peptide values were around 50% higher in the statin group compared to the placebo group [9]. The underlying reason was a large range of C-peptide concentrations among patients at 18 months, varying from 0.1 to 2.3 nmol/l. Because of this apparent heterogeneity we investigated whether baseline patient parameters characterize those with good preservation of beta cell function when treated with atorvastatin from those with less preservation.

There was no association between atorvastatin treatment outcome and age or sex. Of the metabolic parameters analyzed, only liquid-mixed meal stimulated C-peptide levels at baseline was associated with outcome. I.e., patients with above median levels of stimulated C-peptide at baseline exhibited higher stimulated C-peptide levels at 18 months of statin treatment than patients with baseline levels below the median (p<0.031). The same association was observed in the placebo group, suggesting that the association of baseline stimulated C-peptide secretion with C-peptide outcome is independent of atorvastatin treatment. Indeed, it has been reported previously that there is a positive association between the level of C-peptide secretion at diagnosis of type 1 diabetes and residual beta cell function measured in later years [10], [11], [12].

Two major factors modulated by statin treatment are serum cholesterol and CRP. Both, total serum cholesterol and CRP concentrations were lowered significantly by atorvastatin treatment. Other lipid parameters such as LDL-cholesterol, HDL-cholesterol and triglyceride concentrations were also modulated by atorvastatin as expected [9] and therefore were not analyzed separately. However, there was no association between baseline levels of total serum cholesterol and C-peptide outcome, and between the effect of atorvastatin treatment on cholesterol levels and C-peptide outcome.

The positive correlation between baseline CRP concentrations and C-peptide outcome in the patients of the statin group was not seen in those of the placebo group. At baseline, CRP concentrations ranged from 0.08 to 6.34 mg/l in patients of the statin group, and those patients with values above the median of 0.86 mg/l exhibited significantly higher stimulated C-peptide levels at 18 months than those with initially low CRP levels. Interestingly, there was no correlation between the extent of CRP level lowering by atorvastatin and C-peptide outcome. This suggests that increased CRP concentrations at baseline are a marker of responsiveness to atorvastatin treatment, but that the degree of CRP level lowering is not directly involved in the better preservation of beta cell function in response to atorvastatin. Interestingly, an anti-inflammatory CRP-lowering activity of atorvastatin was observed in a study of type 2 diabetes patients only for the subgroup with increased levels of CRP (>2 mg/l) [13].

Most pharmacological effects of statins are due to inhibiting hydroxyl-3-methylglutaryl-coenzyme A reductase. As a consequence, less mevalonate is available for the synthesis of cholesterol. An impaired mevalonate pathway causes lower production of farnesyl or geranylgeranyl pyrophosphates which can modify several transcription factors controlling cell growth, endothelial activity and immune gene expression [14], [15], [16], [17]. At the level of the immune system, statins decreased expression of several pro-inflammatory mediators and receptors [18], [19], [20], [21]. This is accompanied by dampening of aggressive Th1-type and promotion of more benign Th2-type T cell responses [22], [23], [24]. Another relevant target is the endothelium where atorvastatin suppresses inflammatory reactivity and promotes NO bioavailability [25], [26], [27], [28]. It therefore was of interest to compare additional immune characteristics between the subgroup with “high” versus “low” baseline CRP levels. The comparison of serum concentrations of 13 immune mediators indicated significantly higher concentrations of IL-6, IL-1RA, sICAM-1 and E-selectin in the “high” CRP subgroup. For the 10 other pro- or anti-inflammatory immune mediators analyzed, no difference was found between the two subgroups. This indicates that there is no general immune activation in association with increased CRP levels.

Elevated concentrations of IL-6 in the “high” CRP subgroup represent a confirmatory finding because IL-6 is the major internal inductor of CRP production [29] and a correlation between systemic CRP and IL-6 levels has been reported previously [30]. Increased levels of IL-6, IL-1RA, sICAM-1 and E-selection in the “high” CRP group are reminiscent of subclinical systemic inflammation seen in obesity, metabolic syndrome or type 2 diabetes [31], [32], [33], [34]. Hence the “high” CRP subgroup may represent a subgroup among patients with type 1 diabetes which may share some features with type 2 diabetes. Such an interpretation is supported by the observation that patients with “high” CRP levels had a significantly higher BMI and fasting C-peptide concentrations. A positive association of CRP levels with BMI at diagnosis of type 1 diabetes was already noted in a previous study [35].

In conclusion, a higher than median level of plasma CRP at baseline is associated with better preservation of beta cell function in newly diagnosed patients with type 1 diabetes treated with atorvastatin while such an association is not seen in patients in the placebo group. Further analysis of this putative atorvastatin-response subgroup showed characteristics of systemic subclinical inflammation as seen in type 2 diabetes, increased BMI, higher levels of fasting C-peptide and, besides CRP, higher systemic concentrations of IL-6, IL-1RA, sICAM-1 and E-selectin.

## Methods

### Trial procedure and laboratory analyses

In the randomized controlled German multi-center trial DIATOR patients with newly diagnosed type 1 diabetes and at least one islet autoantibody (to glutamic acid decarboxylase 65 or to insulinoma-associated antigen 2, or islet cell antibodies), age 18–39 years received either placebo or atorvastatin for 18 months. The starting atorvastatin dose was 40 mg/d which was increased to 80 mg/d after 4 weeks. For a detailed description of the trial, including the ethics statement see ref. [9].

Stimulated C-peptide secretion was assessed using serum C-peptide concentrations after a standardized liquid mixed meal (Boost HP® (Mead Johnson, Evansville, IN, USA), 6 ml per kg body weight with a maximum of 360 ml) [36]. C-peptide was measured in serum by an immunoenzymatic assay (Biosource/Invitrogen, Karlsruhe, Germany), plasma C-reactive protein (CRP) concentrations were determined by an immunonephelometric assay [37]. All other immune mediators (IL-1RA, IL-6, IL-8, IL-18, IFN-γ, IP-10, MCP-1, MCP-4, MDC, MIP-1β, E-selectin, sICAM-1 and TARC) were determined by double-antibody ELISA or bead-based multiplex technology as described previously [38].

### Statistical Analysis

The statistical analyses were performed with SAS (Version 9.2) and GraphPad Prism (Version 4). Kolmogorov-Smirnov test was performed to determine whether the distribution is normal. For comparison of continuous variables Mann-Whitney *U* test and unpaired *t*-test were used. Association of stimulated C-peptide concentrations at 18 months (dependent variable) with the corresponding baseline parameters were evaluated using multivariate regression models adjusted for age, sex, BMI, fasting C-peptide, stimulated C-peptide, CRP, and IL-6 as appropriate (independent variables; all baseline). Univariate correlation analyses between stimulated C-peptide and total cholesterol or CRP concentrations were performed using Spearman's or Pearson's correlation as appropriate.
